# Diffraction contrast of ferroelectric domains in DPC STEM images

**DOI:** 10.1093/jmicro/dfae019

**Published:** 2024-04-17

**Authors:** Masaya Takamoto, Takehito Seki, Yuichi Ikuhara, Naoya Shibata

**Affiliations:** Institute of Engineering Innovation, School of Engineering, The University of Tokyo, Yayoi 2-11-16, Bunkyo-ku, Tokyo 113-0032, Japan; Institute of Engineering Innovation, School of Engineering, The University of Tokyo, Yayoi 2-11-16, Bunkyo-ku, Tokyo 113-0032, Japan; PRESTO, Japan Science and Technology Agency, Kawaguchi, Saitama 332-0012, Japan; Institute of Engineering Innovation, School of Engineering, The University of Tokyo, Yayoi 2-11-16, Bunkyo-ku, Tokyo 113-0032, Japan; Nanostructures Research Laboratory, Japan Fine Ceramics Center, Mutsuno 2-4-1, Atsuta, Nagoya 456-8587, Japan; Institute of Engineering Innovation, School of Engineering, The University of Tokyo, Yayoi 2-11-16, Bunkyo-ku, Tokyo 113-0032, Japan; Nanostructures Research Laboratory, Japan Fine Ceramics Center, Mutsuno 2-4-1, Atsuta, Nagoya 456-8587, Japan; Quantum-Phase Electronics Center (QPEC), The University of Tokyo, Hongo 7-3-1, Bunkyo-ku, Tokyo 113-8656, Japan

**Keywords:** DPC-STEM, diffraction contrast, elastic scattering, plasmon scattering, ferroelectrics, lithium tantalate

## Abstract

Differential phase contrast scanning transmission electron microscopy (DPC STEM) is a powerful technique for directly visualizing electromagnetic fields inside materials at high spatial resolution. Electric field observation within ferroelectric materials is potentially possible by DPC STEM, but concomitant diffraction contrast hinders the quantitative electric field evaluation. Diffraction contrast is basically caused by the diffraction-condition variation inside a field of view, but in the case of ferroelectric materials, the diffraction conditions can also change with respect to the polarization orientations. To quantitatively observe electric field distribution inside ferroelectric domains, the formation mechanism of diffraction contrast should be clarified in detail. In this study, we systematically simulated diffraction contrast of ferroelectric domains in DPC STEM images based on the dynamical diffraction theory, and clarify the issues for quantitatively observing electric fields inside ferroelectric domains. Furthermore, we conducted experimental DPC STEM observations for a ferroelectric material to confirm the influence of diffraction contrast predicted by the simulations.

## Introduction

Ferroelectric materials are used in many electronic applications such as multilayer ceramic capacitors [1–3] and surface acoustic wave filters [4,5]. The performance of these electronic components severely relies on the ferroelectric domain structures and their response to the applied electric fields. Thus, for the development of advanced electronic components, fundamental understanding of the ferroelectric domain structure and its response to the applied field is essential. Previous studies suggested that the structure and switching behavior of ferroelectric domains are closely linked with charged domain walls and depolarization fields [6–8]. Therefore, to elucidate the fundamental mechanisms governing the ferroelectric domains, it is important to directly characterize the structure, depolarization field and charge distribution within individual ferroelectric domains and domain walls at nanometer dimensions.

**Fig. 1.**
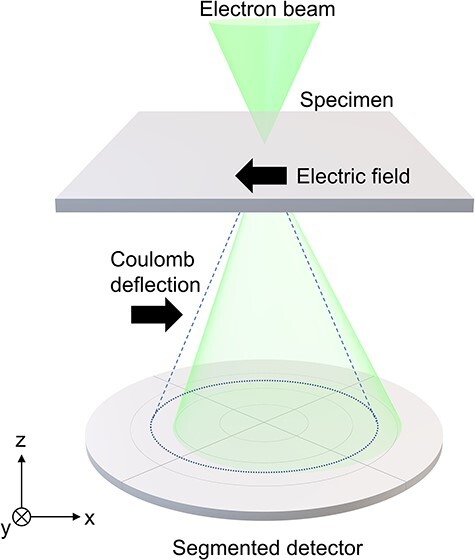
Schematic of DPC STEM. Here, simple electric field case is shown as an example. A segmented detector detects the shift of the bright field disk caused by the Coulomb deflection.

Differential phase contrast scanning transmission electron microscopy (DPC STEM) is a powerful technique that enables direct visualization of electromagnetic fields inside specimens at ultrahigh spatial resolution [9–13]. When focused electron beam is incident in a specimen with electromagnetic fields, the electron beam is deflected by Coulomb or Lorentz forces by the electromagnetic fields (Fig. 1). In DPC STEM, the resultant shift of the bright field (BF) disk on the detector plane is detected by segmented or pixelated detectors. By measuring the magnitude and direction of the shift, the electromagnetic fields can be quantitatively mapped in real space. So far, DPC STEM has enabled real-space observation of *p*–*n* junctions [14], magnetic domains [15,16] and atomic electromagnetic fields [17–20]. In addition, microstructure characterization can be simultaneously carried out by using other STEM techniques such as annular dark field imaging, energy-dispersive X-ray spectroscopy and electron energy-loss spectroscopy.

It is now recognized that DPC images sometimes exhibit strong image contrast not due to the electromagnetic fields, but due to the dynamical diffraction [21–23]. The dynamical diffraction causes nonuniform intensity distribution inside the BF and diffraction disks, resulting in nonzero DPC signals even without electromagnetic fields. This diffraction-induced image contrast is referred to as diffraction contrast and makes it difficult to accurately measure electric fields from DPC STEM images. Therefore, it is crucial to elucidate the formation mechanisms of diffraction contrast in detail and establish the ways to eliminate/minimize such artifacts.

**Fig. 2.**
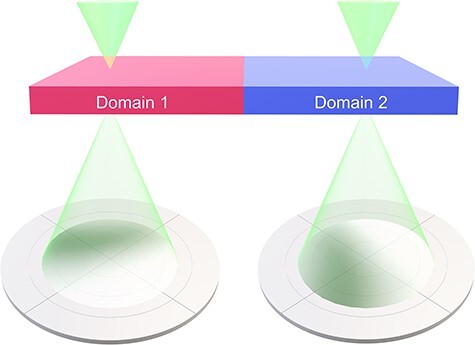
Schematic of DPC STEM in the presence of ferroelectric domains.

The previous DPC STEM observations of *p*–*n* junctions in semiconductors and polycrystalline magnets have shown that the diffraction contrast can be caused by the changes in thickness, specimen bending and crystal misorientation in the field of view [15,23]. In these materials, the electromagnetic fields do not correlate with the crystal structures. Contrastingly, in ferroelectric domains, since the electric fields are expected to correlate with the crystal structures, additional diffraction contrast may also be added. The different polarization directions in ferroelectric domains may alter the diffraction contrast since the slight shift in the position of the atoms due to the different polarization can change the diffraction conditions. Figure 2 shows a schematic illustrating how different ferroelectric domains may change the intensity distribution inside the BF disk, which results in additional diffraction contrast in the DPC image. Therefore, in ferroelectrics, it is more difficult to distinguish the true electric field signal from the diffraction contrast and it is required to investigate the origin of diffraction contrast more in detail.

In this study, we investigated the formation mechanism of diffraction contrast in DPC images caused by different polarization directions. Based on the dynamical diffraction theory, we simulated BF disks with and without inelastic scattering and evaluated the effect of diffraction contrast in detail. Furthermore, the simulation results were experimentally verified by observing the actual diffraction contrast in ferroelectric domains with DPC STEM.

## Investigation of diffraction contrast by simulation

**Fig. 3.**
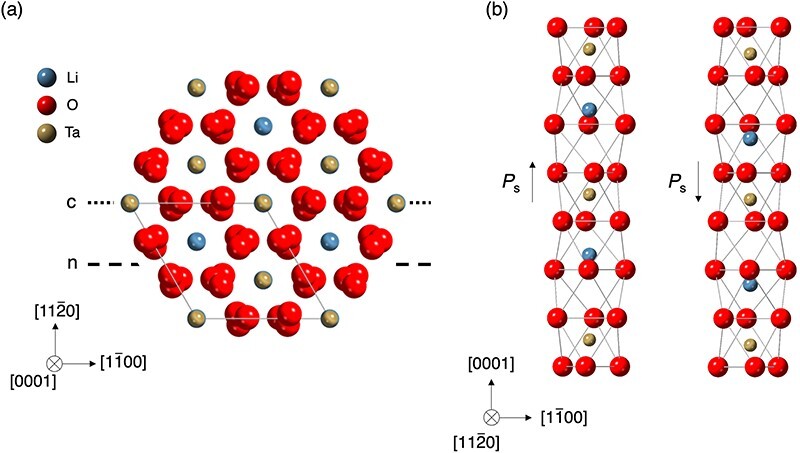
Crystal structure of LiTaO_3_. (a) Projections of the atomic structure along <$0001$>. The dotted line indicates c-glide plane, and the dashed line indicates n-glide plane. (b) The unit cell of +Ps domain and −Ps domain.

In this study, we used lithium tantalate (LiTaO_3_) as a model sample. LiTaO_3_ is a typical trigonal uniaxial ferroelectric with the space group of *R*3*c* [6]. Its Curie temperature, which is the transition temperature from ferroelectric to paraelectric, is ∼690°C, rendering it ferroelectric at ambient temperature [24]. In the ferroelectric phases, the Ta and Li atoms are displaced parallel to the *c*-axis from the center of the oxygen octahedron and the oxygen plane, respectively (Fig. 3). The displacement of the Li and Ta atoms results in the formation of spontaneous polarization (*P*_s_), which can be oriented in only two directions, parallel or antiparallel to the *c*-axis. Therefore, the ferroelectric domains of LiTaO_3_ form two types of 180° domain walls: namely head-to-head and tail-to-tail type domain walls. The 180° domains possess inversion symmetry with each other [6,25].

To investigate the diffraction contrast in DPC images, we simulated convergent-beam electron diffraction (CBED) patterns of LiTaO_3_ based on the dynamical diffraction theory. While the intensity distribution of CBED patterns arises from both elastic and inelastic scattering, the primary contribution to the intensity distribution of BF disk should originate from elastic scattering [26]. At first, our simulations only considered elastic scattering, using the software based on the Bloch-wave dynamical theory of electron diffraction (MBFIT: many-beam dynamical calculations and least-squares fitting [27]). Two different incident beam directions perpendicular to the *c*-axis, <$11\bar 20$> and <$1\bar 100$>, were investigated.

**Fig. 4.**
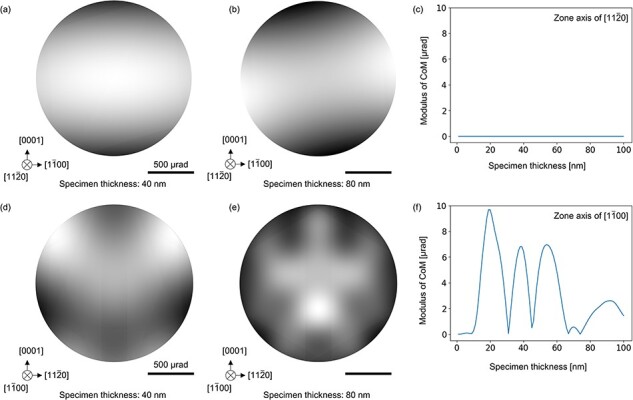
Simulated BF disk patterns for two different sample thicknesses along the zone axis of (a and b) <$11\bar 20$> and (d and e) <$1\bar 100$>. The moduli of CoM of the BF disks at the zone axis of (c) <$11\bar 20$> and (f) <$1\bar 100$> with different sample thicknesses are plotted.

The simulated BF disks with the incident beam directions of <$11\bar 20$> and <$1\bar 100$> are presented in Fig. 4a, b,d and e. The convergence semiangle of the BF disk and acceleration voltage were set to be 852 µrad and 200 kV, which were used in the subsequent experiments. Higher-order Laue zone (HOLZ) reflections were considered up to the first-order Laue zone reflections. The first moments, so-called center of mass (CoM), of the BF disk intensity are plotted in Fig. 4c and f as a function of sample thickness up to 100 nm.

Figure 4 shows that the intensity distribution in the BF disks with the <$11\bar 20$> axis exhibits 2-fold rotation symmetry regardless of the sample thickness (Fig. 4a and b). If the incident beam direction is exactly along the <$11\bar 20$> axis, DPC signal originating from diffraction effect is always zero due to the 2-fold rotation symmetry along the <$11\bar 20$> axis for any sample thickness as shown in Fig. 4c. On the contrary, since the <$1\bar 100$> axis is parallel to the *c*-glide plane as shown in Fig. 3a, the simulated BF disk intensity exhibits mirror symmetry with the *c*-axis serving as the line of symmetry (Fig. 4d and e). Due to the lack of 2-fold rotation symmetry along the <$1\bar 100$> axis, the CoM shows nonzero value and varies with sample thickness as shown in Fig. 4f.

The simulation results suggest that the presence of 2-fold rotation symmetry in the BF disk intensity is important for understanding the diffraction contrast in DPC images. The 2-fold rotation symmetry of diffraction patterns including BF disk can be understood in terms of Friedel’s law. While noncentrosymmetric crystals violate Friedel’s law in whole CBED patterns due to the dynamical diffraction effect [28,29], Friedel’s law appears to remain valid within the simulated BF disk along the <$11\bar 20$> zone-axis of LiTaO_3_. It has been experimentally and theoretically shown that the 2-fold rotation symmetry in the BF disk holds when a mirror or glide plane is present perpendicular to the incident beam direction even for noncentrosymmetric crystals [30]. This is because of the presence of symmetric multiple diffraction paths including HOLZ. In the present case, the intensity distribution is two-fold rotation symmetric due to the presence of *c*-glide and *n*-glide planes perpendicular to the <$11\bar 20$> zone-axis as shown in Fig. 3a. On the contrary, Friedel’s law in the BF disk along the <$1\bar 100$> zone-axis is broken because of the absence of mirror or glide planes perpendicular to the incident beam direction. As a result, the CoM is zero for the <$11\bar 20$> zone-axis case and not for the <$1\bar 100$> zone-axis case.

**Fig. 5.**
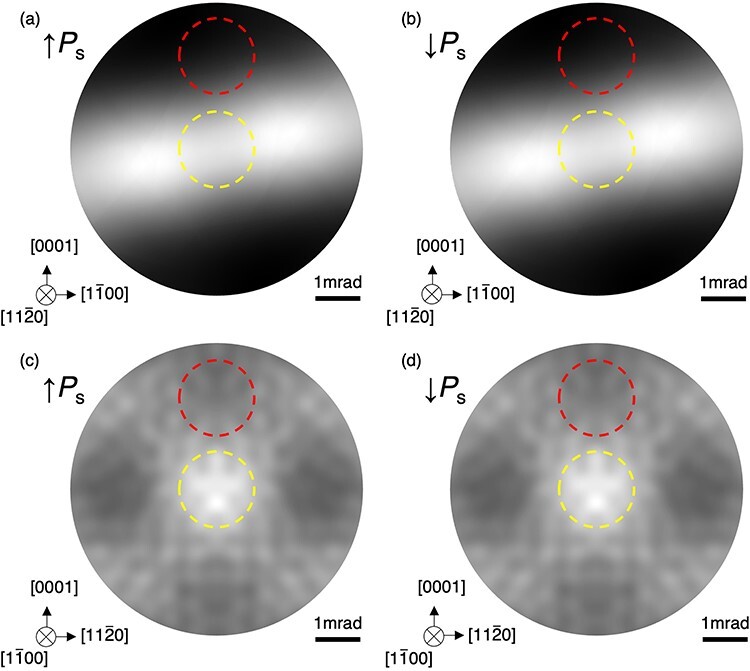
Simulated BF patterns for two different polarization directions along the zone axis of (a and b) <$11\bar 20$> and (c and d) <$1\bar 100$>. The specimen thickness is 80 nm. The lower dashed circles (yellow) represent the radius of 852 µrad at the zone axes, which corresponds to Fig. 4b and e. The upper dashed circles (red) represent the BF disks misoriented by ∼0.1° from the <$11\bar 20$> and <$1\bar 100$> zone-axes to the <$0001$> axis with the radius of 852 µrad.

Next, we examine the difference in diffraction contrast between the adjacent 180° domains. It should be noted that the symmetry operation that transforms the crystal structure of one domain to the other is the inversion symmetry operation, not the 180-degree rotation nor the mirror symmetry operation, because only the inversion symmetry operation is a lattice-invariant operation for the rhombohedral lattice. The diffraction contrast arising from the polarization direction changes were investigated by image simulations. In the simulations, a larger area of BF pattern was simulated to consider the presence of misorientation from the zone axes, since the BF disk with a misorientation can be obtained by cropping the simulated pattern into the circle with the size of the convergence angle. Figure 5a and c shows the BF patterns simulated with the same conditions as those in Fig. 4b and e, respectively, except for the convergence semiangle. Figure 5b and d shows the simulated BF patterns with the opposite polarization. Despite the polarization change, the BF patterns with the opposite polarization are identical to the original patterns (Fig. 5a and c), respectively. For the <$11\bar 20$> incident beam direction, these patterns are 2-fold rotation symmetric due to the presence of the glide planes perpendicular to the incident beam direction as discussed above, and thus the polarization change does not affect the patterns. For the <$1\bar 100$> incident beam direction, 2-fold rotation symmetry in the BF disks is broken due to the absence of a mirror or glide plane perpendicular to the <$1\bar 100$> axis as discussed above. However, the resultant BF patterns with the opposite polarizations are identical as shown in Fig. 5c and d. This is because the 2-fold rotation-symmetry breaking is caused by dynamical diffraction associated with the HOLZ reflections but the geometrical arrangement of the HOLZ reflections does not change with the inversion symmetry operation. While the phase of HOLZ reflections may be changed by the inversion symmetry operation, a multiple diffraction path for the BF disk, ${{\bf{g}}_1} + {{\bf{g}}_2} + {{\bf{g}}_3} + \ldots = 0$, always has the reverse path, $ - {{\bf{g}}_1} - {{\bf{g}}_2} - {{\bf{g}}_3} - \ldots = 0$, and the inversion symmetry operation transforms the one path to the other. Hence, the inversion symmetry operation does not change the diffraction condition and the BF patterns for the opposite polarizations are identical. The yellow and red circles represent the BF disks with the convergence angle of 852 µrad without and with a misorientation. Since the whole patterns are identical for the polarization change, the BF disks are also identical. Therefore, the DPC signal due to the diffraction is not expected to vary between the 180° domains even with some misorientations from the zone axes.

To summarize the investigation on the elastic scattering, <$11\bar 20$> incident beam direction is preferable compared with the <$1\bar 100$> incident beam direction since DPC signal due to diffraction is expected to be zero for the exact <$11\bar 20$> zone-axis. However, in the practical experiment, misorientation from the zone axis is unavoidable due to specimen bending, and thus, DPC signal due to diffraction may be nonzero for the <$11\bar 20$> incident beam direction. However, for the both <$11\bar 20$> and <$1\bar 100$> incident beam directions, the nonzero DPC signals originated from diffraction are the same between the adjacent 180° domains, and only cause uniform intensity backgrounds in the DPC images. Therefore, true electric field signals can be extracted from the DPC images by subtracting such uniform backgrounds across the 180° domains. In conclusion, electric field observations can be performed with the both incident beam directions when inelastic scattering can be negligible.

**Fig. 6.**
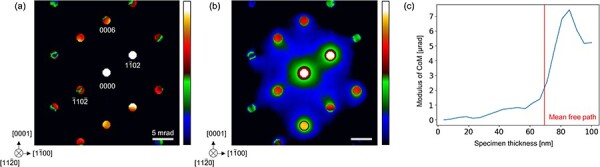
Simulated <$11\bar 20$> CBED patterns of LiTaO_3_ (a) without plasmon scattering and (b) with plasmon scattering. The specimen thickness for those CBED patterns is ∼90 nm. (c) The modulus of CoM for the BF disk and its surroundings with plasmon scattering for the specimen thicknesses ranging from a few nm to 100 nm.

Next, the effect of inelastic scattering is considered. Here, we consider plasmon scattering, which is the dominant inelastic scattering on CBED patterns. We focused specifically on the incident beam direction of <$11\bar 20$>. CBED simulations considering the plasmon scattering were performed using the software package *Dr. Probe* [31]. The simulated CBED patterns with and without plasmon scattering are shown in Fig. 6a and b, where the polarization direction is parallel to the *c*-axis and the convergence semiangle is 852 µrad. The mean free path, critical angle and characteristic angle of LiTaO_3_ required for the CBED simulations is set to be 70 nm, 0.05 mrad and 10 mrad, respectively [32,33].

It is seen that the plasmon scattering blurs the CBED pattern. In the present case, Friedel’s law holds within the BF disk and the CoM is originally zero even with multiple scattering as discussed above. However, the breakdown of Friedel’s law in the diffracted beams can affect the intensity distribution around and inside the BF disk through plasmon scattering. Specifically, $1\bar 102$ and $\bar 110\bar 2$ diffraction disks are located close to the BF disk, and their intensities are significantly different in the present condition. As a result, plasmon scattering via the $1\bar 102$ and $\bar 110\bar 2$ diffraction disks affects the intensity distribution around and inside the BF disk, and therefore the CoM becomes nonzero. To quantitatively investigate this effect, we calculated the CoM inside a circle with the radius of 1.36 mrad, which is slightly larger than the BF disk and the same size as the segmented detector used in the experiments shown later. The calculated CoM modulus for variable specimen thicknesses up to 100 nm is shown in Fig. 6c. While the CoM is close to zero for the specimens sufficiently thinner than the mean free path of the plasmon scattering, CoM can be nonzero for the specimens with a thickness comparable to or thicker than the mean free path.

When the polarization changes in LiTaO_3_, the whole CBED pattern is inverted, resulting in a change in the CoM signal due to the plasmon scattering. Thus, the diffraction contrast due to plasmon scattering can cause additional contrast in DPC STEM images. This plasmon-induced DPC signal is more critical than the DPC signal due to the elastic scattering because the plasmon-induced DPC signal can differ between the ferroelectric domains, and thus causes contrast difference between different domains making very difficult to extract true electric field signals. However, the plasmon-induced diffraction contrast can be suppressed if specimen is thinner than the mean free path of the plasmon scattering or energy filter is applied to cut the plasmon scattering. It is worth noting that optimum specimen thickness must be selected with consideration that the DPC signals are proportional to the sample thickness on condition that the electric field is uniform along the sample thickness. Although the optimum specimen thickness depends on the samples and the magnitude of electric field, the mean free path provides one guideline.

The summary of the above theoretical analysis is as follows. When only considering elastic scattering, the BF disk with the <$11\bar 20$> incident beam direction is 2-fold rotation symmetric while that with <$1\bar 100$> incident beam direction is not. As a result, DPC signal due to diffraction is expected to be zero for the exact <$11\bar 20$> incident beam direction. In the presence of misorientation from the zone axes, DPC signal due to diffraction is expected to be nonzero for the both incident beam directions, but no signal difference is expected between the 180° domains. When the specimen thickness is comparable to or thicker than the mean free path of plasmon scattering, plasmon scattering can cause additional DPC signals. Therefore, DPC STEM observations should be performed with a specimen sufficiently thinner than the mean free path or using an energy filter to eliminate plasmon scattering effects.

## Methods

To confirm the above theoretical expectations of the diffraction contrast in LiTaO_3_, experimental DPC STEM observations were carried out. The TEM specimen was prepared by cutting a LiTaO_3_ single-crystal substrate and thinning it using mechanical polishing and ion milling techniques. Prior to the DPC STEM observations, the ferroelectric domains were observed by conventional dark-field TEM technique with the 0006 diffraction spot using JEM-2100HC (JEOL Ltd.). The observations were performed in the areas where the specimen thickness was both thinner and thicker than the mean free path. The specimen thickness was measured by STEM-EELS. Furthermore, to determine the polarity, we performed CBED observations of the ferroelectric domains and compared the experimental results with simulations. For the CBED observations, the convergence semiangle was 3 mrad. After observing the domains and determining polarity, DPC STEM observations of LiTaO_3_ ferroelectric domains along <$11\bar 20$> direction were performed using JEM-2100 F (JEOL Ltd.), operating at an accelerating voltage of 200 kV. The segment annular all field (SAAF) detector [34] with 16 segments was used. The convergence semiangle was set to be 852 µrad. The BF disk edge was positioned in the middle of the third annulus of the SAAF detector. The DPC STEM observations were carried out at the same areas as the TEM dark-field observations. DPC STEM observations with various sample tilt conditions were performed to verify the effect of diffraction contrast.

## Experimental results

**Fig. 7.**
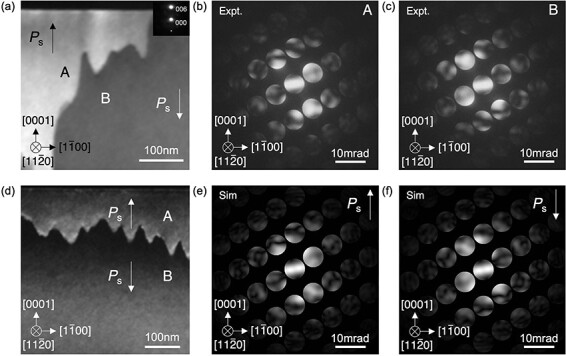
Dark field TEM images of LiTaO_3_ with the specimen thicknesses of about (a) 100 nm and (d) 40 nm, respectively. The diffraction pattern for the TEM dark filed images is shown as an inset in (a). Experimental CBED patterns from the (b) domain A and (c) domain B indicated in (a). (e and f) Simulated CBED patterns with opposite polarization directions along the *c*-axis. The specimen thickness is set to 100 nm. ⊗ represents the direction into the paper.

Figure 7 shows dark-field TEM images and CBED patterns of LiTaO_3_ observed along the <$11\bar 20$> direction. The ferroelectric domains were observed from the regions with the thicknesses of ∼100 nm and 40 nm, which are thicker and thinner than the mean free path, respectively. By comparing the experimental and simulated CBED patterns, it was confirmed that the observed ferroelectric domains are 180° domains and the domain wall is tail-to-tail type.

**Fig. 8.**
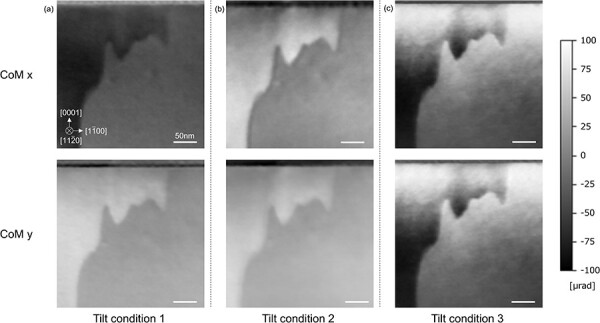
DPC STEM images with various specimen tilt conditions in the area where the specimen thickness exceeds the mean free path of LiTaO_3_. The upper images show CoM *x* and the lower images show CoM *y* images.

Figure 8 shows the DPC STEM images obtained from the sample thickness of ∼100 nm. Image contrast differences were observed between the 180° domains since the sample thickness exceeds the mean free path of the plasmon scattering. Here, several DPC images were acquired with slightly changing specimen-tilt conditions, where the tilt differences are ∼0.1°. It is seen that the image contrast changed drastically as shown in Fig. 8. This indicates that the observed contrast is likely diffraction contrast. Since no diffraction contrast is expected when only elastic scattering is considered, the plasmon-induced diffraction contrast should be the dominant factor in these images.

**Fig. 9.**
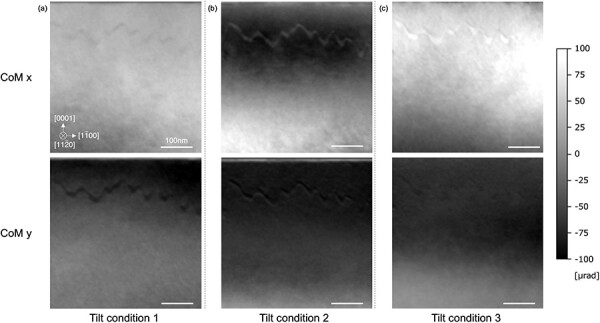
DPC STEM images of LiTaO_3_ with various sample tilt conditions in the area where the specimen thickness is below the mean free path. The upper images show CoM *x* and the lower images show CoM *y* images.

Figure 9 shows the DPC STEM images obtained from the sample area with thickness of ∼40 nm. No significant difference was observed in the DPC signals between the 180° domains. To investigate the diffraction contrast effect in this condition, several DPC images were recorded with changing tilt conditions by ∼0.1°. While the sample tilting changed the contrast across all field of view because of the diffraction contrast caused by the specimen tilt, no apparent DPC signal differences were observed between the 180° domains. Note that the domain wall is visible in the DPC images and the contrast at the domain wall varies with tilt conditions. This may be because diffraction condition at the domain wall differs due to the inclination of the wall or different atomic structures of the wall. To discuss these effects in detail, CBED simulations with a large supercell are required, which will be our future study. In any case, it is thus experimentally confirmed that the sample thickness thinner than the mean free path of the plasmon scattering can be used at least for imaging relative electric field differences between 180° domains of LiTaO_3_.

## Conclusions

In this research, we investigated the formation mechanism of diffraction contrast in DPC STEM images of ferroelectric LiTaO_3_. Our experimental and simulated results indicate that DPC contrast difference between the 180° domains in LiTaO_3_ varies due to the diffraction contrast originated from plasmon scattering. It is noteworthy that the plasmon scattering-derived diffraction contrast becomes considerably stronger when the thickness of the specimen exceeds the mean free path. Thus, plasmon scattering can significantly influence the diffraction contrast in DPC STEM images of ferroelectric domains. To quantitatively observe the electric field distribution within ferroelectric domains using DPC STEM, it is necessary to suppress the diffraction contrast arising from plasmon scattering. One approach to suppressing the effect of plasmon scattering is to reduce the specimen thickness until the plasmon scattering effect becomes negligible. Another approach is to utilize an energy filter to cut off plasmon scattering before detecting electrons by detectors. As for suppressing the diffraction contrast due to elastic scattering, a tilt-scan system for DPC STEM has been reported to be very effective [23,35]. By using these methods to suppress the diffraction contrast arising from both elastic and plasmon scattering, it will become possible to quantitatively estimate true electric fields within ferroelectric domains from DPC STEM images.
